# Remote Baduanjin improves cognition and Hippocampal Connectivity in Subjective Cognitive Decline

**DOI:** 10.1093/geroni/igaf122.3234

**Published:** 2025-12-31

**Authors:** Yuan Feng, Sierra Hodges, Bryn Siegel, Valeria Saccà, Yu Liu, Hana Gross, Bonnie Wong, Jian Kong

**Affiliations:** Massachusetts General Hospital, Boston, Massachusetts, United States; Massachusetts General Hospital, Boston, Massachusetts, United States; Massachusetts General Hospital, Boston, Massachusetts, United States; Massachusetts General Hospital, Boston, Massachusetts, United States; Massachusetts General Hospital, Boston, Massachusetts, United States; Massachusetts General Hospital, Boston, Massachusetts, United States; Massachusetts General Hospital, Boston, Massachusetts, United States; Massachusetts General Hospital, Boston, Massachusetts, United States

## Abstract

Subjective Cognitive Decline (SCD) is gaining attention as a risk factor for Alzheimer’s Disease (AD), representing an optimal intervention stage before irreversible damage occurs. This randomized controlled study examines the effects of Baduanjin (BDJ), a mind-body exercise, on the modulation of cognitive function and its underlying mechanisms in individuals with SCD. A total of 120 SCD participants were randomly assigned to the BDJ group (n = 60) or the cognitive training (CT) group (n = 60) for six months. All interventions were administered remotely. MRI and cognitive assessments including Preclinical Alzheimer’s Cognitive Composite (PACC) and Everyday Cognition scales (ECog) were conducted at baseline, 3 months, and 6 months, with an additional cognitive follow-up at 9 months. Transcriptome-neuroimaging analysis using the Allen Human Brain Atlas explored genetic associations with hippocampal functional connectivity differences. Both groups showed significant PACC improvement at 3 and 6 months (p < 0.001), with no significant group differences. No significant improvement was reported on the ECog in either group. Neuroimaging analysis revealed BDJ training is associated with increased hippocampal connectivity with dorsolateral prefrontal cortex, precentral gyrus, and precuneus from baseline to 6 months compared to CT. Transcriptome-neuroimaging analysis linked group differences in hippocampal functional connectivity to neurotransmitter regulation, synaptic plasticity and neuroinflammation-related pathways. Our findings suggest that on-line BDJ training may modulate hippocampal-cognitive and hippocampal-motor cortical functional connectivity, potentially facilitating cognitive improvement.

